# GWA Study Identifies Two Positive Regulators of Mycotoxin Fumonisin B1 Tolerance in Arabidopsis

**DOI:** 10.3390/genes17030348

**Published:** 2026-03-21

**Authors:** Yaxin Guan, Houpeng Wu, Zhiqing Wang, Chuang Liu, Wangsheng Zhu

**Affiliations:** State Key Laboratory of Maize Bio-Breeding, Ministry of Agriculture and Rural Affairs Key Laboratory of Surveillance and Management for Plant Quarantine Pests, College of Plant Protection, China Agricultural University, Beijing 100193, China

**Keywords:** Fumonisin B1, *Arabidopsis thaliana*, GWAS, toxin tolerance, programmed cell death

## Abstract

Background: Fumonisin B1 (FB1) is a toxic secondary metabolite produced by *Fusarium* species that commonly contaminates cereal crops, posing serious threats to crop productivity and food safety. In plants, FB1 inhibits ceramide synthase, disrupts sphingolipid metabolism, and induces growth inhibition and programmed cell death. Despite the agricultural importance of fumonisin contamination, genetic strategies to enhance FB1 tolerance or detoxification capacity in crops remain limited, largely due to an incomplete understanding of the underlying genetic determinants. Methods: To identify genetic determinants associated with FB1 tolerance, we exploited natural variation in *Arabidopsis thaliana* and conducted a genome-wide association study (GWAS). Candidate genes were further examined using gene expression analyses and functional characterization of overexpression and SALK mutant lines. Results: GWAS revealed a significant association locus on chromosome 1 linked to FB1 tolerance. Two adjacent genes within this locus, *AT1G14750* and *AT1G14755*, were identified as positive regulators of FB1 tolerance. Both genes were rapidly induced upon FB1 exposure. Functional analyses demonstrated that overexpression of either gene significantly enhanced tolerance to FB1-induced damage, whereas SALK mutant lines displayed increased sensitivity, manifested by enhanced growth inhibition and necrosis. Conclusions: Our study identifies *AT1G14750* and *AT1G14755* as previously uncharacterized components of FB1 tolerance in Arabidopsis. These findings provide new insights into the genetic architecture of plant response to mycotoxin stress and establish a foundation for further studies on the molecular mechanisms underlying FB1 tolerance.

## 1. Introduction

Fumonisins are a group of structurally related mycotoxins predominantly produced by *Fusarium verticillioides*, *F. proliferatum*, *F. fujikuroi*, *F. oxysporum*, and related species that infect major cereal crops, particularly maize [1,2,3]. Based on their chemical structures, fumonisins are classified into types A, B, C, and P [4]. Among them, B-type fumonisins (FBs) are the most prevalent, with fumonisin B1 (FB1) being the most abundant and toxic member. FB1 is not only a major contaminant in agricultural products but also widely used as a molecular tool to investigate sphingolipid-mediated programmed cell death (PCD) in plants [5,6,7,8]. FB1 contamination has been reported worldwide, occurring mainly in maize, rice, wheat, and their derived products [9,10]. Accumulation of FB1 adversely affects crop quality and yield. Moreover, due to its well-documented toxicity to humans and animals, including hepatotoxicity, nephrotoxicity, neurotoxicity, and carcinogenicity, FB1 residues represent an important food safety concern [10,11,12].

FB1 exerts its toxicity primarily by inhibiting ceramide synthase, a key enzyme in sphingolipid biosynthesis, leading to the accumulation of free long-chain bases and subsequent disruption of membrane integrity and cellular signaling [7,12]. In plants, FB1 treatment induces hypersensitive response-like cell death accompanied by reactive oxygen species (ROS) accumulation, reprogramming of hormone signaling pathways, and extensive transcriptional changes, making it a valuable tool for studying sphingolipid metabolism and PCD [7,13,14]. In crop species such as maize, FB1 not only contributes to disease severity during *F. verticillioides* infection but also directly impairs plant growth and development, thereby exacerbating yield losses [10,15].

The biosynthetic pathway of FB1 in *Fusarium* species has been extensively characterized. FB1 production is mediated by the fumonisin biosynthetic gene cluster (*FUM*), which comprises 16 genes encoding biosynthetic enzymes and accessory proteins required for fumonisin synthesis [16,17]. Although substantial progress has been made in elucidating the fungal regulatory mechanisms controlling FB1 biosynthesis., genetic studies have demonstrated that disruption of key *FUM* genes, such as *FUM1*, *FUM6*, *FUM8*, and *FUM12*, while severely impairing fumonisin production, does not significantly reduce the incidence of Fusarium ear rot in maize [18,19,20]. These findings suggest that targeting fungal toxin biosynthesis alone may be insufficient to mitigate FB1-associated crop damage.

In plants, studies in *Arabidopsis thaliana* and maize have partially identified several components involved in FB1 perception and downstream signaling, including sphingosine kinases, hormone signaling pathways, and antioxidant systems, which modulate FB1-induced cell death and toxicity [7,13,21,22]. Nevertheless, most of these components were identified through candidate-gene or reverse-genetic approaches. Consequently, a systematic understanding of the natural genetic variation underlying FB1 tolerance in plants remains limited.

Genome-wide association studies (GWASs) provide a powerful framework for dissecting complex traits governed by natural genetic variation [23,24]. By exploiting historical recombination events in diverse populations, GWAS enables high-resolution mapping of loci underlying quantitative traits. In maize, GWAS has been widely applied to identify genes controlling agronomically important traits, including disease and pest resistance. For example, the β-glucosidase gene *ZmBGLU17*, the broad-spectrum disease resistance gene *ZmLecRK1*, and the sheath blight resistance gene *ZmRRS1* were all identified through GWAS-based approaches, highlighting the effectiveness of this strategy for resistance gene discovery [25,26,27]. In *A. thaliana*, GWAS has likewise been successfully employed to uncover loci associated with disease resistance, abiotic stress tolerance, and diverse metabolic traits, providing a robust platform for linking nature variation to gene function [28,29,30,31,32,33]. Nevertheless, despite FB1 being a well-established phytotoxic mycotoxin and a potent inducer of PCD, GWAS-based identification of genetic determinants underlying FB1 tolerance in plants has not been systematically explored.

In this study, we performed a GWAS using a diverse *A. thaliana* population to identify loci associated with FB1 tolerance. We identified a significant locus on chromosome 1 and subsequently prioritized two adjacent candidate genes, *AT1G14750* and *AT1G14755*, located upstream of the lead single-nucleotide polymorphism (SNP). Through genetic and physiological analyses, including overexpression and SALK mutant lines approaches as well as FB1-induced necrosis assays at both whole-plant and leaf levels, we demonstrate that *AT1G14750* and *AT1G14755* act as positive regulators of FB1 tolerance. Collectively, our findings uncover previously uncharacterized components of FB1 response pathways and provide new insights into the genetic architecture underlying sphingolipid-mediated regulation of cell death in plants.

## 2. Methods

### 2.1. Plant Materials and Growth Conditions

*Arabidopsis thaliana* accessions used for Genome-wide association study (GWAS) were obtained from publicly available natural population resources. *A. thaliana* Columbia-0 (Col-0) was used as the wild-type control in all functional analyses. Plants were grown under short-day conditions (8 h light/16 h dark) at 22 °C with a light intensity of approximately 120 μmol m^−2^ s^−1^ and relative humidity of 60%. For plate-based assays, seeds were surface sterilized and germinated on ½ MS medium. For Fumonisin B1 (FB1) infiltration and gene expression analysis, plants were grown in soil for 4 weeks prior to treatment.

### 2.2. Genome-Wide Association Analysis of FB1 Tolerance

FB1 tolerance phenotypes of *Arabidopsis* natural accessions were used for GWAS. For phenotypic evaluation, seeds were germinated and grown on medium supplemented with 0.4 μM FB1 under controlled growth conditions (16 h light/8 h dark, 22 °C) for 10 days. FB1 tolerance was quantified based on seedling survival rates, defined as the ratio of surviving seedlings to the total number of seedlings, and used as the phenotypic trait for association mapping. A total of 124 *A. thaliana* accessions were included in the analysis (Table S1).

SNP genotype data were obtained from 1001 Genomes Project. Association analysis was performed using the easyGWAS platform with the efficient mixed-model association (EMMAX) algorithm to account for population structure and kinship [34]. SNPs were filtered using a minor allele frequency (MAF) threshold of ≥0.04. Of the initial 6,973,565 SNPs, 2,149,382 SNPs passed quality filtering and were retained for further analysis. Genome-wide significance was determined using a Bonferroni correction (α = 0.05), corresponding to a threshold of *p* = 2.33 × 10^−8^. Manhattan and quantile–quantile (Q-Q) plots were generated to visualize association signals and assess model performance.

### 2.3. Candidate Gene Identification and SNP Annotation

Significant SNPs identified by GWAS were annotated using the Variant Effect Predictor (VEP) via Ensembl REST services based on the *A. thaliana* TAIR10 reference genome. Genomic locations, predicted variant consequences, and distances to nearby genes were determined. Candidate genes were selected based on their physical proximity to lead SNPs. Genes located immediately upstream or downstream of the peak SNP on chromosome 1 were prioritized for further analyses.

### 2.4. FB1 Treatment and Tissue Sampling

FB1 was dissolved in DMSO and infiltrated into fully expanded rosette leaves of 4-week-old plants using a needleless syringe. An equivalent volume of DMSO was used as a mock control. For time-course experiments, leaves were infiltrated with 8 μM FB1, and samples were collected at 0, 6, 12, 24, 48, and 72 h post infiltration. At each time point, tissues were harvested from infiltrated leaf areas, immediately frozen in liquid nitrogen, and stored at −80 °C until further processing. For phenotypic analysis, plants were maintained under controlled growth conditions (16 h light/8 h dark, 22 °C) after infiltration. Leaves were harvested 5 d after FB1 treatment, photographed, and used for phenotypic evaluation and quantification of FB1-induced damage.

### 2.5. RNA Extraction and RT-qPCR

Total RNA was extracted using an RNA isolator Total RNA Extraction Reagent (R401, Vazyme Biotech, Co. Ltd., Nanjing, China). cDNA was synthesized from 1 μg of high-quality total RNA (2.0 > A260/A280 > 1.8), using HiScript III first-stand cDNA synthesis (R312-01, Vazyme Biotech). RT-qPCR was performed with a ChamQ Universal SYBR qPCR master mix (Q711, Vazyme Biotech) in a Thermo Fisher system (ABI Quant-Studio 6 Flex, Thermo Fisher Scientific, USA). Gene-specific primers were used to amplify *AT1G14750* and *AT1G14755*. Expression levels were normalized to *ACTIN2* (*AT3G18780*), and relative transcript abundance was calculated using the 2^−ΔΔCt^ method [35]. Primers are listed in Table S2.

### 2.6. Bioinformatic Analysis of AT1G14750 and AT1G14755

The amino acid sequence of AT1G14750 and AT1G14755 were retrieved from TAIR database. Signal peptide prediction was performed using SignalP 5.0 (https://services.healthtech.dtu.dk/services/SignalP-5.0/, accessed on 15 March 2026) with default parameters. Homologous protein sequences from representative plant species were obtained from public databased based on sequence similarity searches. Multiple sequence alignment was conducted using Jalview (version: 2.11.5.1) with default parameters. Conserved regions were visualized using Jalview, and domain features were annotated based on sequence alignment and known protein family characteristics.

### 2.7. Generation of Transgenic Plants

The coding sequences of *AT1G14750* and *AT1G14755* were amplified from Col-0 cDNA and cloned into the entry vector pUC19 using a homologous recombination-based cloning system (ClonExpress Ultra One Step Cloning Kit, C115, Vazyme Biotech Co. Ltd., Nanjing, China). The resulting constructs were subsequently transferred into the binary vector pCAMBIA1300, which contains a CaMV 35S promoter to drive gene expression and a hygromycin resistance marker for plant selection. The pCAMBIA1300 vector was obtained from our laboratory collection. All constructs were generated following the method described by Wang et al. [36]. The constructs were introduced into Col-0 plants using the floral dip method. Transgenic plants were advanced to the T2 generation, from which homozygous lines were identified. At least two independents homozygous T2 lines for each construct were selected and propagated, and T3 seeds derived from these lines were used for subsequent phenotypic and molecular analyses.

### 2.8. Quantification and Statistical Analysis

The data for the quantification analyses are expressed as means ± SEM. To perform statistical analyses, GraphPad Prism software (version 10.0) and Microsoft Excel 2021 were utilized.

## 3. Results

### 3.1. Differential Resistance to Fumonisin B1 Among Arabidopsis thaliana Accessions

**Figure 1:**
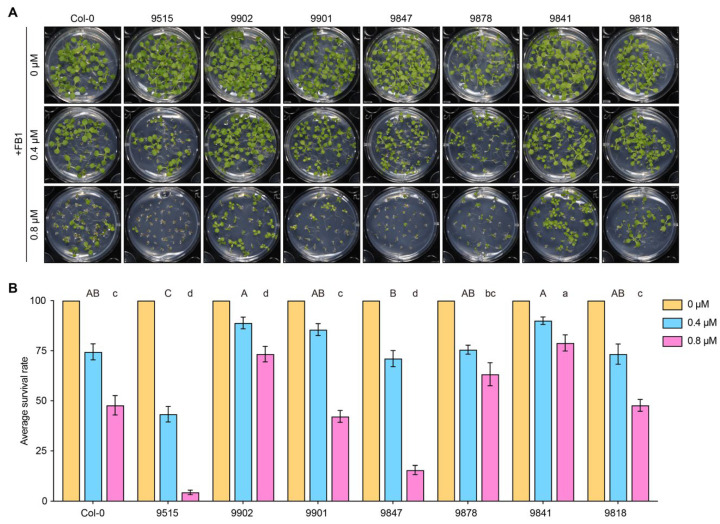
Natural variation in fumonisin B1 (FB1) tolerance among *Arabidopsis thaliana* ecotypes. (**A**): Representative seedling phenotypes of eight Arabidopsis ecotypes (Col-0, 9515, 9902, 9901, 9847, 9878, 9841, and 9818) grown on medium supplemented with 0, 0.4, or 0.8 μM FB1. Surface-sterilized seeds were germinated and grown under identical conditions, and images were taken at the seedling stage. Ecotypes displayed distinct growth responses to FB1 treatment. The numbers above each panel indicate ecotype identifiers. FB1 concentrations are indicated on the left. (**B**): Quantification of FB1 tolerance based on seedling survival rates. Seedlings were classified as surviving or dead according to their growth phenotypes, and average survival rates were calculated for each ecotype under the indicated FB1 concentrations. Data represent means ± SEM. Different letters indicate statistically significant differences among ecotypes under the same treatment (One-way ANOVA followed by Tukey’s multiple comparison test, *p* < 0.05). Color codes represent FB1 concentrations: yellow (0 μM), blue (0.4 μM), and pink (0.8 μM).

To assess natural variation in *Arabidopsis thaliana* ecotypes in response to FB1, surface-sterilized seeds of different ecotypes were sown on medium supplemented with 0.4 μM or 0.8 μM FB1, with an equal volume of DMSO included as a solvent control. Under control conditions, all tested ecotypes displayed normal germination and seedling growth. In contrast, FB1 treatment resulted in ecotype-dependent differences in seedling growth responses (Figure 1A).

We randomly selected eight *A. thaliana* ecotypes, including Col-0, and assessed their tolerance to FB1. Ecotypes 9902, 9901, and 9841 exhibited robust growth on medium containing 0.4 μM FB1, indicating relatively high FB1 tolerance, whereas ecotype 9515 showed severely compromised growth under the same conditions, indicative of strong FB1 sensitivity (Figure 1A). At 0.8 μM FB1, ecotypes 9902 and 9841 remained highly tolerant (Figure 1A). To quantitatively assess FB1 tolerance, plants were classified as surviving or dead based on their growth phenotypes (Supplementary Figure S1), and survival rates were calculated for each ecotype. Survival analysis of the eight ecotypes revealed significant natural variation in FB1 tolerance at both 0.4 μM and 0.8 μM FB1 concentrations (Figure 1B). These results indicate that natural variation among *Arabidopsis* ecotypes can be leveraged to identify genetic determinants underlying FB1 tolerance.

### 3.2. Genome-Wide Association Analysis Identifies Loci Associated with FB1 Tolerance in Arabidopsis thaliana

**Figure 2:**
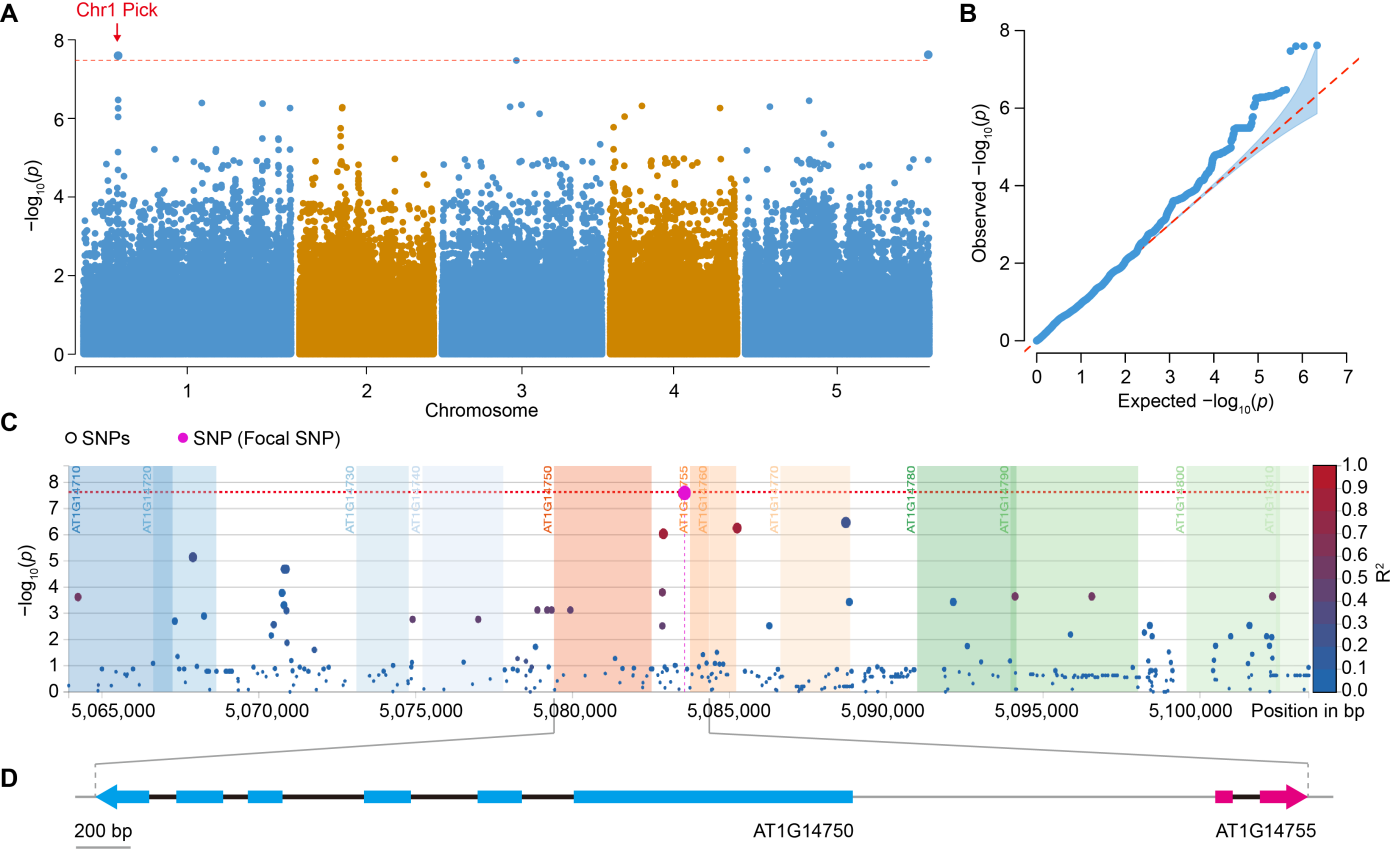
GWAS identifies a candidate locus for FB1 tolerance in Arabidopsis (**A**): Manhattan plot showing genome-wide association results for FB1 tolerance across 124 Arabidopsis ecotypes. The horizontal red line indicates the Bonferroni-corrected significance threshold (*p* = 2.33 × 10^−8^). The arrow marks the lead SNP on chromosome 1. (**B**): Quantile–quantile (QQ) plot of observed versus expected log_10_(*p*) values, indicating appropriate control of population structure and inflation. (**C**): Regional association plot for the candidate locus on chromosome 1. SNPs are colored according to linkage disequilibrium (R^2^) with the lead SNP (purple). (**D**): Schematic representation of gene structures for *AT1G14750* and *AT1G14755* surrounding the lead SNP. The relative position of the association signal is indicated. Blue squares represent exon of *AT1G14750*, red squares represent exons of *AT1G14755*, black lines represent introns, gray lines represent the *A. thaliana* genome, arrows indicate gene coding direction, bar = 200 bp.

GWA study revealed several loci associated with FB1 tolerance, with the most prominent association peak detected on chromosome 1 (Figure 2A). This peak exceeded the genome-wide significance threshold, and the lead SNP was located at position 5,083,572 bp. Quantile–quantile analysis showed that the observed *p* value distribution closely followed the expected distribution except for highly significant SNPs, indicating effective control of population structure and minimal inflation (Figure 2B).

To further resolve the candidate locus, regional association analysis was conducted for the Chr1: 5.06–5.10 Mb interval. The lead SNP was in linkage disequilibrium with several neighboring SNPs, forming a distinct association block (Figure 2C). Annotation of this region revealed that the lead SNP is located in an intergenic region, with the closest genes being *AT1G14750* and *AT1G14755*. The lead SNP is positioned 1036 bp upstream of *AT1G14750* and 104 bp upstream of *AT1G14755* (Table 1). The Variant Effect Predictor (VEP) annotation further supports this, indicating that the SNP is a modifier variant that may influence gene expression but not protein sequence (Table 1). To further confirm the physical relationship between the association signal and the candidate genes, the gene structures of *AT1G14750* and *AT1G14755* were visualized (Figure 2D). The lead SNP is located in the upstream regulatory region of *AT1G14755*, which is in close proximity to *AT1G14750*, further suggesting their potential involvement in FB1 tolerance.

Allelic distribution analysis revealed that the lead SNP consists of a major allele (C/C) and a major allele (G/G) in the natural population (Supplementary Figure S2A). Phenotypic analysis showed that ecotypes carrying the different alleles exhibited distinct FB1 tolerance phenotypes, with the G/G genotype correlating with lower survival rates under FB1 treatment (Supplementary Figure S2B). These findings suggest that *AT1G14750* and *AT1G14755* are strong candidate genes for FB1 tolerance, and the lead SNP may influence FB1 tolerance through regulatory mechanisms at these loci.

### 3.3. FB1 Treatment Induces Expression of Candidate Genes AT1G14750 and AT1G14755

To investigate whether the candidate genes identified by GWAS respond to FB1 treatment in planta, we analyzed the expression of *AT1G14750* and *AT1G14755* in Arabidopsis leaves after 8 μM FB1 infiltration. Arabidopsis plants were grown under short-day conditions for one month, and fully expanded leaves were infiltrated with 8 μM FB1. Leaf tissues were collected at defined time points (0, 6, 12, 24, 48, and 72 h post-infiltration) for RT-qPCR analysis.

**Figure 3:**
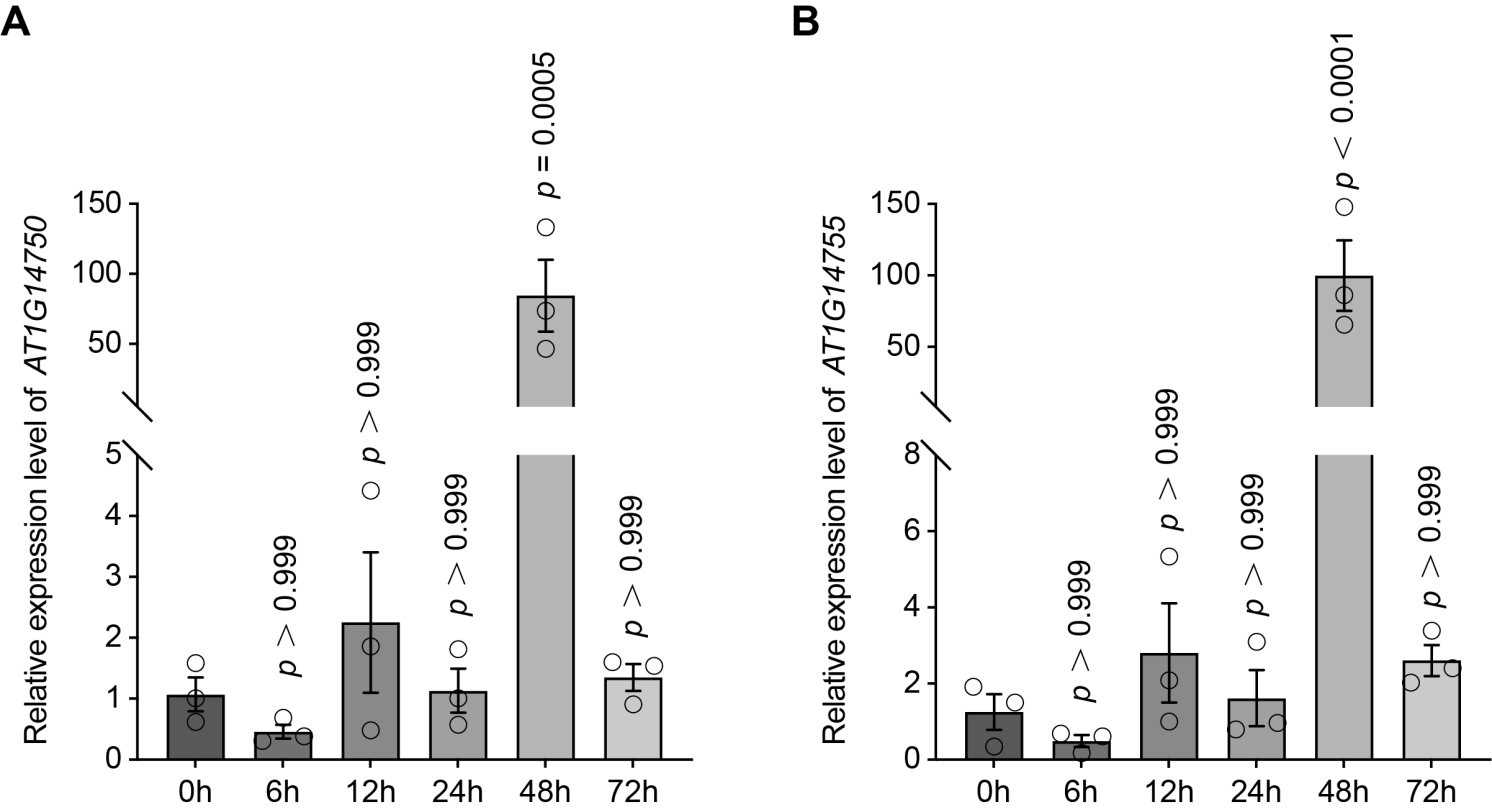
FB1-induced expression dynamics of candidate genes *AT1G14750* and *AT1G14755*. (**A**): Relative expression levels of *AT1G14750* in Arabidopsis seedlings following FB1 treatment at the indicated time points (0, 6, 12, 24, 48, and 72 h). (**B**): Relative expression levels of *AT1G14755* in Arabidopsis seedlings following FB1 treatment at the indicated time points (0, 6, 12, 24, 48, and 72 h). Open circles represent individual biological replicates.

Both *AT1G14750* and *AT1G14755* exhibited similar temporal expression patterns in response to FB1. Transcript levels of both genes remained relatively stable during the early phase of treatment (0–24 h), showing no significant induction compared with the 0 h (prior to FB1 treatment) (Figure 3A,B). In contrast, a pronounced increase in expression was observed for both genes at 48 h post-infiltration. *AT1G14750* displayed a strong induction at 48 h (*p* = 0.0005), while *AT1G14755* showed an even more robust response (*p* < 0.0001). At 72 h, transcript levels of both genes decreased and returned to levels comparable to the 0 h (Figure 3A,B). The coordinated late induction of *AT1G14750* and *AT1G14755* in response to FB1 treatment suggests that these neighboring genes may be co-regulated and participate in FB1-associated stress responses in Arabidopsis.

### 3.4. Bioinformatic Analysis Suggests Distinct Molecular Features of AT1G14750 and AT1G14755

**Figure 4:**
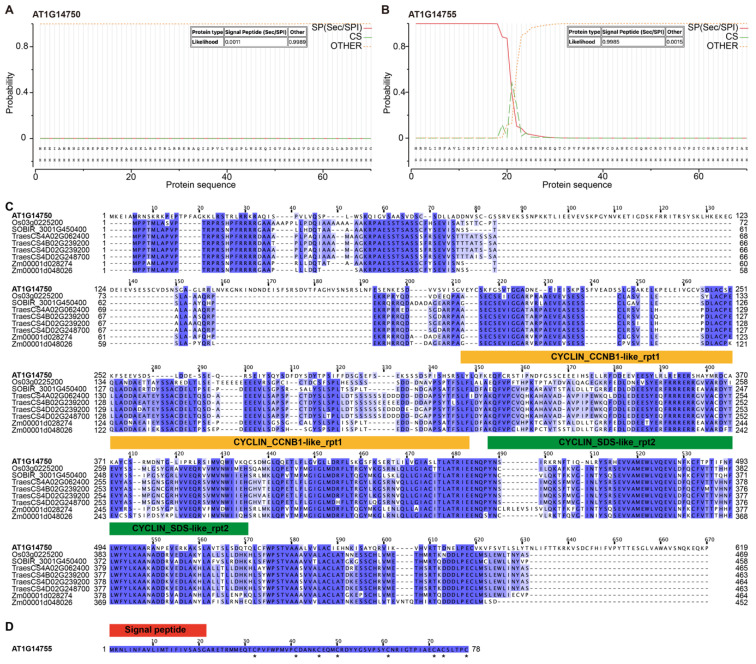
Bioinformatic characterization and sequence analysis of AT1G14750 and AT1G14755. (**A**,**B**): Prediction of N-terminal signal peptides in AT1G14750 (**A**) and AT1G14755 (**B**) using SignalP 5.0. The probability of signal peptide (SP), cleavage site (CS), and non-secretory regions (OTHER) along the protein sequences is shown. AT1G14755 is predicted to contain a signal peptide, whereas AT1G14750 is not. (**C**): Multiple sequence alignment of AT1G14750 with homologous proteins from representative plant species. Conserved regions are highlighted in blue. Predicted conserved domains related to cyclin-like protein families (CYCLIN_CCNB1-like_rpt1 and CYCLIN_SDS-like_rpt2) are indicated. (**D**): Amino acid sequence of the N-terminal region of AT1G14755 showing the predicted signal peptide. Asterisks indicate conserved cysteine residues.

To gain insight into the potential molecular functions of AT1G14750 and AT1G14755, we performed a series of bioinformatic analyses on their encoded protein sequences. Signal peptide prediction indicated that AT1G14755 contains a putative N-terminal signal peptide, whereas AT1G14750 lacks any detectable signal peptide (Figure 4A,B). Consistently, Gene Ontology (GO) annotations obtained from the TAIR database indicated that AT1G14750 is mainly localized to the nucleus and cytoplasm, while AT1G14755 is predicted to be localized to the extracellular region (Table S3). These results suggest that the two proteins may function in distinct cellular compartments, with AT1G14750 likely involved in intracellular regulatory processes and AT1G14755 potentially participating in extracellular or membrane-associated signaling events related to stress responses.

Multiple sequence alignment revealed that AT1G14750 is highly conserved across different plant species and contains several conserved regions (Figure 4C). Notably, domain analysis identified sequence features related to cyclin-like protein families, including CYCLIN_CCNB1-like and CYCLIN_SDS-like motifs. These features suggest that AT1G14750 may be involved in regulatory processes such as cell cycle progression or stress-induced cellular reprogramming. In contrast, AT1G14755 displays distinct structural characteristics. Signal peptide prediction indicated the presence of an N-terminal signal peptide, supporting its secretion into the extracellular space (Figure 4B and Table S3). Consistently, sequence analysis further revealed that the mature AT1G14755 peptide contains eight conserved cysteine residues, forming a typical cysteine-rich domain characteristic of plant defensin-like (DEFL) proteins. Given these properties, AT1G14755 is likely to function in extracellular signaling, including stress perception, intercellular communication, or modulation of the extracellular environment under FB1-induced stress conditions. Taken together, these results indicate that AT1G14750 and AT1G14755 possess distinct molecular features and may operate through different mechanisms. This functional divergence may underlie their coordinated yet potentially complementary roles in mediating FB1 tolerance in Arabidopsis.

### 3.5. AT1G14750 and AT1G14755 Positively Regulate FB1 Tolerance in Arabidopsis

To functionally validate the role of the GWAS-identified candidate genes *AT1G14750* and *AT1G14755* in FB1 tolerance, we independently generated overexpression lines (OE1 and OE2) for each gene in the Col-0 background and purchased the SALK mutant lines for each gene (Supplementary Figure S3). Under control conditions (+DSMO), neither SALK mutants nor overexpression lines of *AT1G14750* and *AT1G14755* displayed obvious differences in seedling growth or morphology compared with Col-0 (Figure 4A,B and Supplementary Figure S3A,C), indicating that altered expression of these genes does not substantially affect plant development in the absence of FB1 stress.

**Figure 5:**
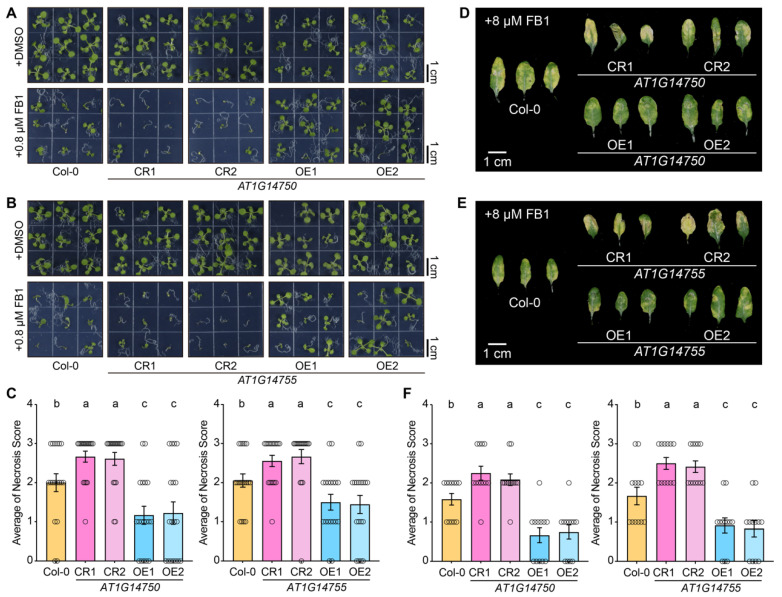
Functional validation of *AT1G14750* and *AT1G14755* in FB1 tolerance. (**A**): Seedling phenotypes of Col-0, *AT1G14750* SALK mutants (CR) and overexpression lines (OE) grown on ½ MS medium supplemented with DMSO (control) or 0.8 μM FB1. (**B**): Seedling phenotypes of Col-0, *AT1G14755* SALK mutants (CR) and overexpression lines (OE) under control or FB1 treatment conditions. (**C**): Quantification of FB1-induced necrosis in seedlings shown in (**A**,**B**). Necrosis severity was scored on a 0–3 scale, and the average necrosis score is presented. (**D**): Representative leaf phenotypes of four-week-old Col-0, *AT1G14750* SALK mutants, and overexpression lines following infiltration with 8 μM FB1. (**E**): Representative leaf phenotypes of Col-0, *AT1G14755* SALK mutants, and overexpression lines after 8 μM FB1 infiltration. (**F**): Quantification of necrosis severity in FB1-infiltrated leaves shown in (**D**,**E**). Data in (**C**,**F**) are presented as means ± SEM (*n* ≥ 12). Individual data points are shown as open circles. Different lowercase letters indicate statistically significant differences among genotypes (One-way ANOVA followed by Tukey’s multiple comparison test, *p* < 0.05). Scale bars = 1 cm.

When grown on a medium supplemented with 0.8 μM FB1, Col-0 seedlings exhibited pronounced growth inhibition and necrotic symptoms. In contrast, *AT1G14750* and *AT1G14755* overexpression lines displayed markedly enhanced FB1 tolerance, characterized by improved seedling growth and reduced necrosis (Figure 5A,B). Conversely, SALK mutants in both genes showed more severe growth arrest and necrotic symptoms than Col-0 (Figure 5A,B). Quantification of FB1-induced damage using necrosis scoring revealed that overexpression lines of both *AT1G14750* and *AT1G14755* had significantly lower average necrosis scores than Col-0, whereas SALK mutants exhibited significantly higher scores (Figure 5C, Supplemental Figure S4A). These results indicate that both genes act as positive regulators of FB1 tolerance at the seedling stage.

To further assess FB1 responses in mature tissues, fully expanded leaves of four-week-old plants were infiltrated with 8 μM FB1. Consistent with the seedling assays, leaves of *AT1G14750* and *AT1G14755* overexpression lines developed substantially fewer necrotic lesions than those of Col-0, whereas SALK mutants displayed enhanced lesion formation and tissue collapse (Figure 5D,E). Necrosis scoring confirmed a significant reduction in FB1-induced damage in overexpression lines and an increase in SALK mutants relative to Col-0 (Figure 5F and Supplementary Figure S4B). Taken together, these results demonstrate that *AT1G14750* and *AT1G14755* positively regulate FB1 tolerance in Arabidopsis, functioning to mitigate FB1-induced necrosis at both seedling and mature leaf stages.

## 4. Discussion

In this study, we exploited natural variation in *Arabidopsis thaliana* to dissect the genetic basis of tolerance to FB1, an agriculturally relevant mycotoxin. Pronounced differences in FB1 sensitivity among Arabidopsis ecotypes indicate that FB1 tolerance is a quantitatively inherited trait rather than a uniform species-wide response (Figure 1). Importantly, all ecotypes were evaluated under identical growth conditions, and FB1-specific effects were evident at concentrations that had minimal impact on control plants, suggesting that the observed variation reflects differential FB1 responses rather than general growth vigor.

By integrating GWAS with functional validation, we identified a major locus on chromosome 1 and demonstrated that *AT1G14750* and *AT1G14755* act as positive regulators of FB1 tolerance (Figure 2 and Figure 5). The lead SNP was located in the intergenic region between these two genes (Figure 2C,D and Table 1), raising the possibility that either or both contribute to the association signal. Consistent with this, independent overexpression of each gene significantly enhanced FB1 tolerance, whereas SALK mutant lines exhibited increased sensitivity (Figure 5). The convergence of GWAS signals and reciprocal phenotypes in overexpression and SALK mutant lines strongly supports a causal role for both genes in modulating FB1 tolerance, although coordinated regulation within this genomic region cannot be excluded.

The FB1-induced transcriptional responses of *AT1G14750* and *AT1G14755* further suggest that these genes participate in inducible stress-response pathways rather than constitutive detoxification mechanisms (Figure 3). Their dynamic expression patterns, characterized by delayed but pronounced induction, imply involvement in adaptive responses to toxin-induced cellular perturbation rather than immediate perception events.

Bioinformatic analyses provide additional insight into the potential molecular functions of these two genes. *AT1G14750* encodes a conserved protein containing cyclin-like domains, including motifs related to CYCLIN_CCNB1 and CYCLIN-SDS family proteins, and it predicted to localize to the nucleus and cytoplasm (Figure 4A,C and Table S3). In plants, cyclins function as key regulatory subunits of cyclin-dependent kinases and are central to cell cycle progression, checkpoint regulation, and stress adaptation [37,38,39]. Increasing evidence indicates that cell cycle regulators are tightly linked to stress responses, as environmental stresses frequently trigger cell cycle arrest and reprogramming to maintain cellular homeostasis [40,41]. Notably, plant-specific cyclins such as SOLO DANCERS (SDS) have been implicated in specialized regulatory processes beyond canonical mitotic control, highlighting the functional diversification of cyclin proteins [42,43]. Given that FB1 disrupts sphingolipid metabolism and induces PCD, the cyclin-like features of AT1G14750 suggest that it may act as a regulatory component coordinating cell cycle-associated checkpoints or cellular recovery processes under toxin stress. Such a role is consistent with the enhanced sensitivity observed in SALK mutant lines and the increased tolerance conferred by overexpression.

In contrast, *AT1G14755* encodes a defensin-like (DEFL) protein with a predicted N-terminal signal peptide, indicating secretion or extracellular localization (Figure 4B,D and Table S3). Plant defensin and defensin-like peptides are small cysteine-rich proteins widely implicated in antimicrobial defense, stress signaling, and cell–cell communication [44,45]. Beyond their classical roles in inhibiting microbial growth, DEFL proteins have been shown to function as signaling molecules in developmental processes and stress responses. Based on these properties, AT1G14755 is unlikely to function directly in intracellular metabolic pathways such as sphingolipid biosynthesis. Instead, it may contribute to FB1 tolerance by modulating extracellular stress perception, amplifying defense-related signaling, or influencing the local extracellular environment during toxin exposure. Although DEFL family proteins are widely distributed in plants [46], the apparent absence of clear AT1G14755 homologs in major crops suggests functional divergence or lineage-specific specialization (Figure 4D).

Together, these observations suggest that AT1G14750 and AT1G14755 possess distinct molecular features and may contribute to FB1 tolerance through different yet potentially complementary mechanisms, linking intracellular regulatory processes with extracellular stress signaling. Although their genomic proximity and similar expression patterns raise the possibility of coordinated regulation (Figure 2D and Figure 3), further genetic and biochemical analyses will be required to determine whether they function in the same pathway or act independently.

Previous studies on plant responses to FB1 have largely relied on candidate gene or reverse genetic approaches, focusing on defined components of PCD, sphingolipid metabolism, and hormone signaling [12,13,14]. Although these studies have provided important mechanistic insights, they do not capture the contribution of naturally occurring allelic variation to FB1 tolerance. Our findings complement this body of work by demonstrating that natural variation at specific loci can substantially influence whole-plant responses to FB1, highlighting the value of GWAS for uncovering regulators that may be overlooked by hypothesis-driven strategies.

Notably, FB1 tolerance appears to be a polygenic trait, and the locus identified here likely represents one component of a broader genetic architecture. Additional loci with smaller effect sizes may remain undetected under stringent multiple-testing correction. Increasing population size, refining phenotypic resolution, or integrating multi-trait and multi-omics approaches may further resolve the genetic basis of FB1 tolerance.

Although this study was conducted in *A. thaliana*, our findings have important implications for crop improvement. The conservation of AT1G14750 homologs across major cereal crops, including rice, maize, wheat, and sorghum, suggests that this gene family may represent a promising target for enhancing tolerance to Fumonisin contamination in agriculturally important species. In contrast, the apparent absence of AT1G14755 homologs in these crops highlights potential divergence in FB1 response mechanisms between model plants and crops, underscoring the importance of comparative functional analyses. These results suggest that both conserved regulators and lineage-specific components contribute to plant responses to mycotoxin stress.

In summary, our study demonstrates that natural variation-based mapping, coupled with targeted functional analyses, is an effective strategy for identifying regulators of FB1 tolerance in plants. The identification of *AT1G14750* and *AT1G14755* as positive regulators advances our understanding of plant responses to mycotoxin stress and provides a genetic framework for future studies aimed at improving tolerance to Fumonisin-associated damage.

## 5. Conclusions

In this study, we identified genetic loci associated with natural variation in FB1 tolerance in *Arabidopsis thaliana* through a GWAS and characterized two adjacent genes, *AT1G14750* and *AT1G14755*, as positive regulators of FB1 tolerance. Both genes were induced by FB1 treatment, and functional analyses demonstrated that overexpression enhanced tolerance, whereas SALK mutant lines exhibited increased sensitivity. Bioinformatic analyses further suggested that these two genes possess distinct molecular features, implying that they may function through different mechanisms in response to FB1 stress. Collectively, our findings provide new insights into the genetic basis of plant responses to mycotoxin stress and identify candidate genes that may contribute to improving stress tolerance and crop resilience.

## Figures and Tables

**Table 1:** Variant Effect Predictor (VEP) Effects of the Pick SNP (Chr1: 5,083,572 bp).

Consequence	Impact	Variant Allele	Distance	Strand	Gene ID
upstream gene variant	Modifier	G	104	+	*AT1G14755*
upstream gene variant	Modifier	G	1036	−	*AT1G14750*

“+” and “−” indicate the forward (sense) and reverse (antisense) DNA strands, respectively.

## Data Availability

The original contributions presented in this study are included in the article and Supplementary Materials. Further inquiries can be directed towards the corresponding authors.

## Supplementary Materials

The following supporting information can be downloaded at: https://www.mdpi.com/article/10.3390/genes17030348/s1, Figure S1: Criteria for classification of seedling survival and death under FB1 treatment; Figure S2: Allelic distribution and phenotypic association of the lead SNP on chromosome 1; Figure S3: Validation of *AT1G14750* and *AT1G14755* overexpression and knockout lines; Figure S4: Scoring criteria for FB1-induced necrosis in *Arabidopsis thaliana*; Table S1: The list of 124 Arabidopsis ecotypes used in GWAS. Table S2: Oligonucleotide primers. Table S3: Gene Ontology (GO)-based subcellular localization annotations of *AT1G14750* and *AT1G14755* obtained from the TAIR database.

## Abbreviations

The following abbreviations are used in this manuscript:

FBs	Fumonisins
FB1	Fumonisin B1
PCD	Programmed cell death
ROS	Reactive oxygen species
FUM	Fumonisin biosynthetic gene cluster
GWAS	Genome-wide association study
SNP	Single-nucleotide polymorphism
EMMAX	Efficient mixed-model association
MAF	Minor allele frequency
Q-Q	Quantile–quantile
VEP	Variant effect predictor
DEFL	Defensin-like
