# Supplementary material for: GWA Study Identifies Two Positive Regulators of Mycotoxin Fumonisin B1 Tolerance in Arabidopsis
Source: Genes (Basel). 2026 Mar 21;17(3):348. doi: 10.3390/genes17030348 (PMC13026401; doi:10.3390/genes17030348)
Supplement: Supplementary file 1 [file genes-17-00348-s001.zip › genes-4210671-supplementary.pdf]

genes

MDPI

GWA Study Identifies Two Positive Regulators of Mycotoxin Fumonisin B1 Tolerance in Arabidopsis

Yaxin Guan [1], Houpeng Wu [1], Zhiqing Wang [1], Chuang Liu [1] and Wangsheng Zhu [1,*]

[1] State Key Laboratory of Maize Bio-breeding/College of Plant Protection/Ministry of Agriculture and Rural Affairs Key Laboratory of Surveillance and Management for Plant Quarantine Pests, China Agricultural University, 100193, Beijing, P.R. China.

* Correspondence: wangshengzhu@cau.edu.cn (W. Z.)

Supplementary File

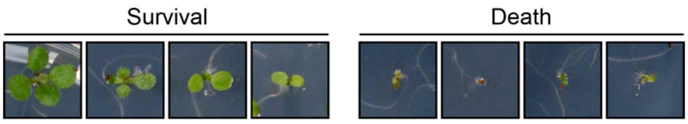

Supplementary Figure S1. Criteria for classification of seedling survival and death under FB1 treatment.

Representative images illustrating the phenotypic criteria used to classify Arabidopsis seedlings as surviving or dead following FB1 treatment. Surviving seedlings exhibited green cotyledons and continued post-germination growth, whereas dead seedlings showed severe growth arrest. Survival rate was calculated as the number of surviving seedlings divided by the total number of seedlings. These criteria were used for survival rate quantification in FB1 tolerance assays.

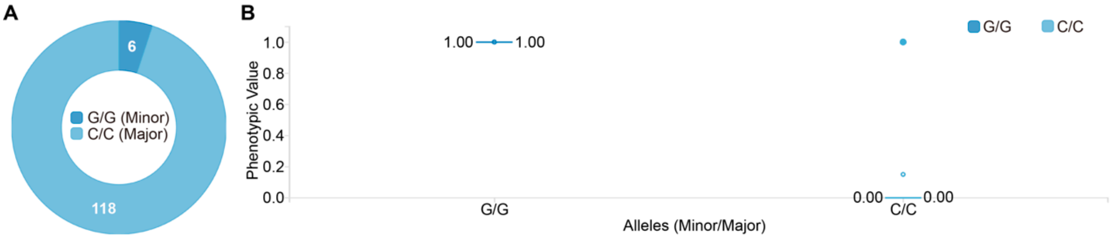

Supplementary Figure S2. Allelic distribution and phenotypic association of the lead SNP on chromosome 1.

A: Allele frequency distribution of the lead SNP (Chr1: 5,083,572) among the 124 *Arabidopsis thaliana* ecotypes used for GWAS analysis. The major allele (C/C) and minor allele (G/G) are indicated, with numbers representing the count of ecotypes carrying each genotype.

B: Association between SNP genotypes and FB1 tolerance phenotypes. Phenotypic values are plotted for ecotypes carrying the minor (G/G) or major (C/C) allele. Each dot represents an individual ecotype, and horizontal lines indicate mean phenotypic values for each genotype group.

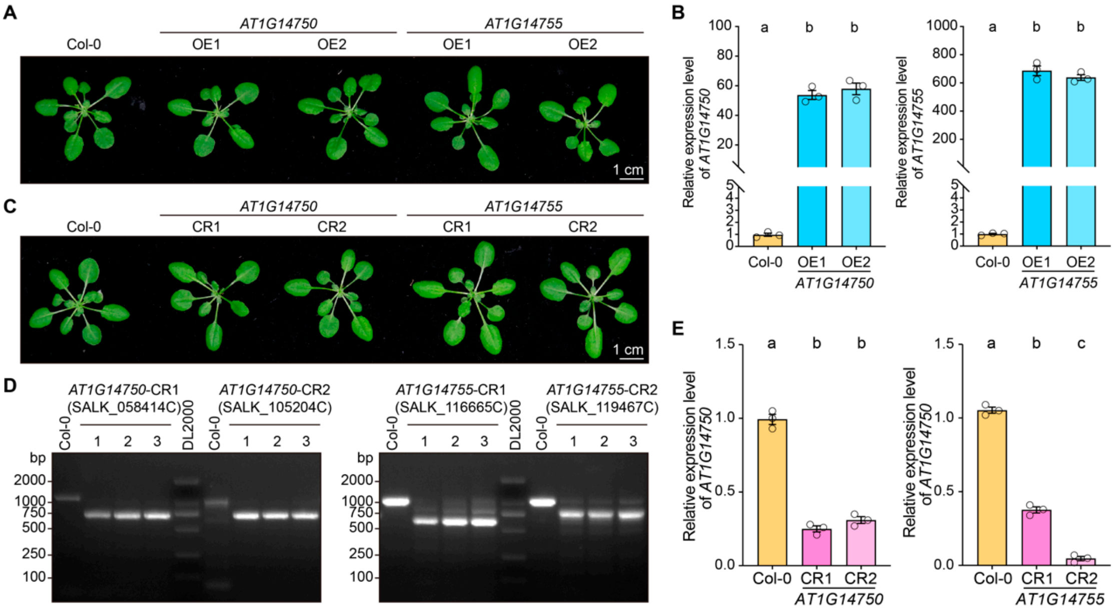

Supplementary Figure. S3 Validation of *AT1G14750* and *AT1G14755* overexpression and SALK mutant lines.

A: Representative rosette phenotypes of wild-type Col-0 and overexpression lines (OE1 and OE2) of *AT1G14750* and *AT1G14755* grown under normal conditions. Scale bars = 1 cm.

B: Relative expression levels of *AT1G14750* and *AT1G14755* in overexpression lines, as determined by RT-qPCR. Transcript levels were normalized to an internal reference gene and are shown relative to Col-0. Bars represent the mean ± SEM of three biological replicates, with individual data points shown as open circles. Different letters indicate statistically significant differences ($P < 0.05$).

C: Representative rosette phenotypes of wild-type Col-0 and SALK lines (CR1 and CR2) of *AT1G14750* and *AT1G14755* under control conditions. Scale bars = 1 cm.

D: Genotyping analysis confirming the identity of T-DNA insertion or genome editing events in the corresponding mutant lines.

E: Relative transcript levels of *AT1G14750* and *AT1G14755* in SALK mutants determined by RT-qPCR, confirming reduced expression compared with Col-0.

Bars in B and E represent the mean ± SEM of three biological replicates, with individual data points shown as open circles. Different letters indicate statistically significant differences ($P < 0.05$).

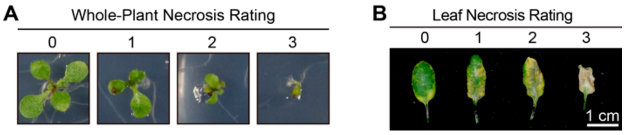

Supplementary Figure. S4 Scoring criteria for FB1-induced necrosis in *Arabidopsis thaliana*.

A: Representative images illustrating the whole-plant rating used for FB1 tolerance assays in seedlings. Necrosis severity was scored on a scale of 0-3: 0, no visible necrosis; 1, mild chlorosis or limited necrotic lesions; 2, moderate necrosis affecting most tissue; 3, severe necrosis or plant death.

B: Representative images showing the leaf necrosis rating scale used for FB1 infiltration assays in mature leaves. Scores range from 0 (no visible necrosis) to 3 (severe necrosis covering most of the leaf area). Scale bar = 1 cm.

Table. S1 The list of 124 Arabidopsis ecotypes used in GWAS

ID	Name	CS_Number	Collector	Survival rate	2IID
4958	UKSW06-360	https://abrc.osu.edu/stocks/number/CS78814	Eric Holub	30%	0
5151	UKSE06-325	https://abrc.osu.edu/stocks/number/CS78801	Eric Holub	30%	0
5349	UKSE06-639	https://abrc.osu.edu/stocks/number/CS78807	Eric Holub	56%	1
5811	UKID107	https://abrc.osu.edu/stocks/number/CS78778	Eric Holub	8%	0
6008	Duk	https://abrc.osu.edu/stocks/number/CS76824	Jirina Relichov	8%	0
6040	Kni-1	https://abrc.osu.edu/stocks/number/CS76970	Magnus Nordborg	8%	0
6105	T450	https://abrc.osu.edu/stocks/number/CS77297	Mattias Jakobsson	30%	0
6106	T460	https://abrc.osu.edu/stocks/number/CS77298	Mattias Jakobsson	30%	0
6115	T580	https://abrc.osu.edu/stocks/number/CS77306	Mattias Jakobsson	30%	0
6128	T740	https://abrc.osu.edu/stocks/number/CS77313	Mattias Jakobsson	31%	0
6131	T780	https://abrc.osu.edu/stocks/number/CS77315	Mattias Jakobsson	57%	1
6133	T800	https://abrc.osu.edu/stocks/number/CS77317	Mattias Jakobsson	8%	0
6148	T960	https://abrc.osu.edu/stocks/number/CS77325	Mattias Jakobsson	0%	0
6169	TÅD 01	https://abrc.osu.edu/stocks/number/CS77333	Mattias Jakobsson	8%	0
6172	TÅD 04	https://abrc.osu.edu/stocks/number/CS77335	Mattias Jakobsson	32%	0
6194	TDr-8	https://abrc.osu.edu/stocks/number/CS77355	Mattias Jakobsson	8%	0
6958	Ra-0	https://abrc.osu.edu/stocks/number/CS76582	Albert Kranz	9%	0
7008	Benk-1	https://abrc.osu.edu/stocks/number/CS76447	Maarten Koornneef	33%	0
7025	Bl-1	https://abrc.osu.edu/stocks/number/CS76450	Albert Kranz	0%	0
7026	Boot-1	https://abrc.osu.edu/stocks/number/CS76452	Maarten Koornneef	67%	1
7028	Bch-1	https://abrc.osu.edu/stocks/number/CS76444	Albert	69%	1

			Kranz		
7071	Chat-1	https://abrc.osu.edu/stocks/number/CS76463	Maarten Koornneef	33%	0
7072	Chi-0	https://abrc.osu.edu/stocks/number/CS76464	Albert Kranz	33%	0
7106	Dr-0	https://abrc.osu.edu/stocks/number/CS78897	Albert Kranz	0%	0
7117	El-0	https://abrc.osu.edu/stocks/number/CS76479	Albert Kranz	60%	1
7126	Es-0	https://abrc.osu.edu/stocks/number/CS76484	Albert Kranz	33%	0
7162	Hs-0	https://abrc.osu.edu/stocks/number/CS76515	Albert Kranz	35%	0
7165	Hn-0	https://abrc.osu.edu/stocks/number/CS76513	Albert Kranz	35%	0
7244	Mnz-0	https://abrc.osu.edu/stocks/number/CS76552	Albert Kranz	35%	0
7282	Or-0	https://abrc.osu.edu/stocks/number/CS76568	Albert Kranz	50%	1
7287	Ove-0	https://abrc.osu.edu/stocks/number/CS76569	Albert Kranz	50%	1
7305	Pt-0	https://abrc.osu.edu/stocks/number/CS78915	Albert Kranz	50%	1
7316	Rhen-1	https://abrc.osu.edu/stocks/number/CS78916	Maarten Koornneef	36%	0
7346	Sten-0	https://abrc.osu.edu/stocks/number/CS77277	Albert Kranz	0%	0
7353	Tha-1	https://abrc.osu.edu/stocks/number/CS76611	Maarten Koornneef	79%	1
8230	Algutsrum	https://abrc.osu.edu/stocks/number/CS76657	Magnus Nordborg	0%	0
8237	Kävlinge-1	https://abrc.osu.edu/stocks/number/CS76964	Torbjorn Sall	0%	0
8242	Lillö-1	https://abrc.osu.edu/stocks/number/CS77039	Magnus Nordborg	38%	0
8243	PHW-2	https://abrc.osu.edu/stocks/number/CS77173	Paul Williams	38%	0
8264	Bla-1	https://abrc.osu.edu/stocks/number/CS76451	Albert Kranz	0%	0
8311	In-0	https://abrc.osu.edu/stocks/number/CS78903	Albert Kranz	0%	0
8343	Na-1	https://abrc.osu.edu/stocks/number/CS76558	Albert Kranz	0%	0
9121	Bak-5	https://abrc.osu.edu/stocks/number/CS76685	James	0%	0

			Beck		
9332	Bar 1	https://abrc.osu.edu/stocks/number/CS76688	Alison Anastasio	39%	0
9336	Bön 1	https://abrc.osu.edu/stocks/number/CS76715	Alison Anastasio	11%	0
9339	Böt 1	https://abrc.osu.edu/stocks/number/CS76720	Alison Anastasio	60%	1
9363	EdJ 2	https://abrc.osu.edu/stocks/number/CS76833	Alison Anastasio	39%	0
9394	Hag-2	https://abrc.osu.edu/stocks/number/CS76907	Alison Anastasio	39%	0
9399	Hamm-1	https://abrc.osu.edu/stocks/number/CS76910	Alison Anastasio	0%	0
9508	IP-Mos-1	https://abrc.osu.edu/stocks/number/CS77108	Carlos Alonso-Blanco	41%	0
9511	IP-Vav-0	https://abrc.osu.edu/stocks/number/CS78835	Carlos Alonso-Blanco	41%	0
9514	IP-Adm-0	https://abrc.osu.edu/stocks/number/CS76647	Carlos Alonso-Blanco	0%	0
9515	IP-Ala-0	https://abrc.osu.edu/stocks/number/CS76650	Carlos Alonso-Blanco	0%	0
9520	IP-Ara-4	https://abrc.osu.edu/stocks/number/CS76670	Carlos Alonso-Blanco	12%	0
9527	IP-Cad-0	https://abrc.osu.edu/stocks/number/CS76739	Carlos Alonso-Blanco	13%	0
9530	IP-Car-1	https://abrc.osu.edu/stocks/number/CS76742	Carlos Alonso-Blanco	14%	0
9535	IP-Coc-1	https://abrc.osu.edu/stocks/number/CS76776	Xavier Picó	14%	0
9541	IP-Fue-2	https://abrc.osu.edu/stocks/number/CS76871	Carlos Alonso-Blanco	14%	0
9561	IP-Mun-0	https://abrc.osu.edu/stocks/number/CS77114	Carlos Alonso-Blanco	15%	0
9562	IP-Mur-0	https://abrc.osu.edu/stocks/number/CS77115	Xavier Picó	15%	0

9567	IP-Pal-0	https://abrc.osu.edu/stocks/number/CS77159	Xavier Picó	15%	0
9577	IP-Ria-0	https://abrc.osu.edu/stocks/number/CS77216	Xavier Picó	43%	0
9578	IP-Sac-0	https://abrc.osu.edu/stocks/number/CS77229	Carlos Alonso-Blanco	43%	0
9587	IP-Tdc-0	https://abrc.osu.edu/stocks/number/CS77344	Carlos Alonso-Blanco	74%	1
9589	IP-Tor-1	https://abrc.osu.edu/stocks/number/CS77378	Carlos Alonso-Blanco	44%	0
9590	IP-Trs-0	https://abrc.osu.edu/stocks/number/CS77387	Carlos Alonso-Blanco	15%	0
9591	IP-Vad-0	https://abrc.osu.edu/stocks/number/CS78826	Carlos Alonso-Blanco	15%	0
9593	IP-Vaz-0	https://abrc.osu.edu/stocks/number/CS78836	Carlos Alonso-Blanco	15%	0
9595	IP-Vdt-0	https://abrc.osu.edu/stocks/number/CS78838	Carlos Alonso-Blanco	7%	0
9609	Adam-1	https://abrc.osu.edu/stocks/number/CS76645	0	16%	0
9610	Lesno-4	https://abrc.osu.edu/stocks/number/CS77034	0	0%	0
9612	Lesno-2	https://abrc.osu.edu/stocks/number/CS77033	0	16%	0
9616	Krazo-1	https://abrc.osu.edu/stocks/number/CS76984	0	17%	0
9619	Basta-1	https://abrc.osu.edu/stocks/number/CS76691	0	17%	0
9625	Kolyv-2	https://abrc.osu.edu/stocks/number/CS76977	0	17%	0
9630	K-oze-3	https://abrc.osu.edu/stocks/number/CS76958	0	18%	0
9631	Lebja-1	https://abrc.osu.edu/stocks/number/CS77015	0	45%	0
9636	Noveg-1	https://abrc.osu.edu/stocks/number/CS77131	0	55%	1
9638	Noveg-3	https://abrc.osu.edu/stocks/number/CS77133	0	46%	0
9649	Bivio-1	https://abrc.osu.edu/stocks/number/CS76713	0	56%	1
9656	Marti-1	https://abrc.osu.edu/stocks/number/CS77072	0	79%	1
9660	Sarno-1	https://abrc.osu.edu/stocks/number/CS77236	0	48%	0
9663	Teano-1	https://abrc.osu.edu/stocks/number/CS77357	0	0%	0
9705	Choto-1	https://abrc.osu.edu/stocks/number/CS76769	0	55%	1
9713	Stara-1	https://abrc.osu.edu/stocks/number/CS77271	0	0%	0
9720	Malak-1	https://abrc.osu.edu/stocks/number/CS77064	0	48%	0
9725	Epidauros-1	https://abrc.osu.edu/stocks/number/CS76844	0	18%	0
9726	Faneronemi-	https://abrc.osu.edu/stocks/number/CS76853	0	8%	0

	3				
9738	Bran-1	https://abrc.osu.edu/stocks/number/CS76722	0	0%	0
9743	Furni-1	https://abrc.osu.edu/stocks/number/CS76873	0	19%	0
9744	Iasi-1	https://abrc.osu.edu/stocks/number/CS76944	0	25%	0
9747	Zabar-1	https://abrc.osu.edu/stocks/number/CS78870	0	19%	0
9749	Knjas-1	https://abrc.osu.edu/stocks/number/CS76971	0	19%	0
9758	Altai-5	https://abrc.osu.edu/stocks/number/CS76433	0	0%	0
9762	Etna-2	https://abrc.osu.edu/stocks/number/CS76487	0	9%	0
9766	Westkar-4	https://abrc.osu.edu/stocks/number/CS76629	0	8%	0
9774	Alt-1	https://abrc.osu.edu/stocks/number/CS76663	0	0%	0
9779	Bai-10	https://abrc.osu.edu/stocks/number/CS76682	0	0%	0
9781	Kus2-2	https://abrc.osu.edu/stocks/number/CS76990	0	0%	0
9795	Wank-2	https://abrc.osu.edu/stocks/number/CS78852	0	0%	0
9803	Muh-2	https://abrc.osu.edu/stocks/number/CS77113	0	21%	0
9821	IP-Aru-0	https://abrc.osu.edu/stocks/number/CS76674	Xavier Picó	21%	0
9826	IP-Bor-0	https://abrc.osu.edu/stocks/number/CS76717	Carlos Alonso-Blanco	73%	1
9834	IP-Cho-0	https://abrc.osu.edu/stocks/number/CS76768	Carlos Alonso-Blanco	64%	1
9841	IP-Ees-0	https://abrc.osu.edu/stocks/number/CS76836	Carlos Alonso-Blanco	0%	0
9867	IP-Mie-1	https://abrc.osu.edu/stocks/number/CS77083	Xavier Picó	62%	1
9869	IP-Moj-0	https://abrc.osu.edu/stocks/number/CS77105	Xavier Picó	63%	1
9878	IP-Pee-0	https://abrc.osu.edu/stocks/number/CS77167	Carlos Alonso-Blanco	0%	0
9879	IP-Per-0	https://abrc.osu.edu/stocks/number/CS77169	Carlos Alonso-Blanco	26%	0
9883	IP-Piq-0	https://abrc.osu.edu/stocks/number/CS77179	Carlos Alonso-Blanco	50%	1
9885	IP-Prd-0	https://abrc.osu.edu/stocks/number/CS77189	Carlos Alonso-Blanco	26%	0
9886	IP-Pru-0	https://abrc.osu.edu/stocks/number/CS77190	Xavier Picó	50%	1
9894	IP-Sen-0	https://abrc.osu.edu/stocks/number/CS77243	Xavier	0%	0

9897	IP-Smt-1	https://abrc.osu.edu/stocks/number/CS77257	Picó Xavier Picó	3%	0
9898	IP-Som-0	https://abrc.osu.edu/stocks/number/CS77259	Carlos Alonso-Blanco	64%	1
9901	IP-Urd-1	https://abrc.osu.edu/stocks/number/CS78824	Carlos Alonso-Blanco	65%	1
9902	IP-Usa-0	https://abrc.osu.edu/stocks/number/CS78825	Carlos Alonso-Blanco	30%	0
9903	IP-Val-0	https://abrc.osu.edu/stocks/number/CS78829	Carlos Alonso-Blanco	30%	0
9920	DIR-9	https://abrc.osu.edu/stocks/number/CS76796	0	6%	0
9927	ARR-17	https://abrc.osu.edu/stocks/number/CS76673	0	6%	0
9928	BEZ-9	https://abrc.osu.edu/stocks/number/CS76703	0	7%	0
9941	Fei-0	https://abrc.osu.edu/stocks/number/CS76412	—	7%	0
10006	Kastel-1	https://abrc.osu.edu/stocks/number/CS76395	James Beck	7%	0
10020	Jl-2	https://abrc.osu.edu/stocks/number/CS76956	—	7%	0

Table. S2 Oligonucleotide primers.

**Primer**	**Sequence**	**Purpose**
YW-30169F	GAATTCAAAGCGCAAGCCTG	RT-qPCR *AT1G14750*
YW-30195R	CGAACCACAGGAAACGTTGT	RT-qPCR *AT1G14750*
YW-30175F	GCTGTTCTTATCATGACCAT	RT-qPCR *AT1G14755*
YW-30176R	ACATGGTGTAAGACTACAGG	RT-qPCR *AT1G14755*
YW-73F	GCCATCCAAGCTGTTCTCTC	RT-qPCR *ACTIN2*
YW-74R	GCTCGTAGTCAACAGCAACAA	RT-qPCR *ACTIN2*
YW-30204F	GATTCAACCAAACCATGAACG	Genotyping of SALK_058414C, *AT1G14750*-CR1, LP
YW-30205R	TTCGAGCTCTTCTCGACTCTG	Genotyping of SALK_058414C, *AT1G14750*-CR1, RP
YW-30206F	TAGGCTGGATTTTGTGACCTG	Genotyping of SALK_105204C, *AT1G14750*-CR2, LP
YW-30207R	CTAGAGAGTGGGCTGATGCAG	Genotyping of SALK_105204C, *AT1G14750*-CR2, RP

YW-30208F	TTCCTCCGAAGCTACCTCTTC	Genotyping of SALK_116665C, *AT1G14755*-CR1, LP
YW-30209R	CATCTTCATCGTTTCAGCCTC	Genotyping of SALK_116665C, *AT1G14755*-CR1, RP
YW-30210F	TTTCTTGCATCAAATCACGTG	Genotyping of SALK_119467C, *AT1G14755*-CR2, LP
YW-30211R	CCATCATTCTCGTCTCTCTCG	Genotyping of SALK_119467C, *AT1G14755*-CR2, RP
LBb1.3	ATTTTGCCGATTTCGGAAC	LBb1.3
YW-4923F	CAAGGCCATTACGGCCatgaaggagatcgcgatg	Generation of 35S::AT1G14750-Flag backbone
YW-4924R	ATTGGCCGAGGCGGCCCCgggtttttgttctttttg	Generation of 35S::AT1G14750-Flag backbone
YW-4927F	CAAGGCCATTACGGCCatgagaaacctcataaat	Generation of 35S::AT1G14755-Flag backbone
YW-4928R	ATTGGCCGAGGCGGCCCCacatggtgtaagactaca	Generation of 35S::AT1G14755-Flag backbone

Table. S3 Gene Ontology (GO)-based subcellular localization annotations of *AT1G14750* and *AT1G14755* obtained from the TAIR database.

Locus	Relationship Type	Keyword	Reference
AT1G14750	Involved in	G1/S transition of mitotic cell cycle	Annotation inferences using phylogenetic trees.
AT1G14750	Enables	Cyclin-dependent protein serine/threonine kinase regulator activity	Annotation inferences using phylogenetic trees.
AT1G14750	Located in	Cytoplasm, nuclues	Annotation inferences using phylogenetic trees.
AT1G14755	Involved in	Biological process	GO_REF:0000015: Use of the ND evidence code for Gene Ontology (GO) terms.
AT1G14755	Enables	Molecular function	GO_REF:0000015: Use of the ND evidence code for Gene Ontology (GO) terms.
AT1G14755	Located in	Extracellular region	AtSubP analysis.
